# Formation of Nitrogen Doped Titanium Dioxide Surface Layer on NiTi Shape Memory Alloy

**DOI:** 10.3390/ma14061575

**Published:** 2021-03-23

**Authors:** Michał Tarnowski, Justyna Witkowska, Jerzy Morgiel, Witold Jakubowski, Bogdan Walkowiak, Tomasz Borowski, Tadeusz Wierzchoń

**Affiliations:** 1 Faculty of Materials Science and Engineering, Warsaw University of Technology, 141 Wołoska St., 02-507 Warsaw, Poland; justyna.aleksandra.witkowska@gmail.com (J.W.); tomasz.borowski@pw.edu.pl (T.B.); tadeusz.wierzchon@pw.edu.pl (T.W.); 2 Polish Academy of Sciences, Institute of Metallurgy and Materials Science, 25 Reymonta St., 30-059 Cracow, Poland; j.morgiel@imim.pl; 3 Institute of Materials Science and Engineering, Lodz University of Technology, 1/15 Stefanowskiego St., 90-924 Lodz, Poland; witold.jakubowski@p.lodz.pl (W.J.); bogdan.walkowiak@p.lodz.pl (B.W.)

**Keywords:** NiTi alloy, glow discharge oxidation, structure, antibacterial properties

## Abstract

NiTi shape memory alloys are increasingly being used as bone and cardiac implants. The oxide layer of nanometric thickness spontaneously formed on their surface does not sufficiently protect from nickel transition into surrounding tissues, and its presence, even in a small amount, can be harmful to the human organism. In order to limit this disadvantageous phenomenon, there are several surface engineering techniques used, including oxidation methods. Due to the usually complex shapes of implants, one of the most prospective methods is low-temperature plasma oxidation. This article presents the role of cathode sputtering in the formation of a titanium dioxide surface layer, specifically rutile. The surface of the NiTi shape memory alloy was modified using low-temperature glow discharge plasma oxidation processes, which were carried out in two variants: oxidation using an argon + oxygen (80% vol.) reactive atmosphere and the less chemically active argon + air (80% vol.), but with a preliminary cathode sputtering process in the Ar + N_2_ (1:1) plasma. This paper presents the structure (STEM), chemical composition (EDS, SIMS), surface topography (optical profilometer, Atomic Force Microscopy—AFM) and antibacterial properties of nanocrystalline TiO_2_ diffusive surface layers. It is shown that prior cathodic sputtering in argon-nitrogen plasma almost doubled the thickness of the produced nitrogen-doped titanium dioxide layers despite using air instead of oxygen. The (TiO_x_N_y_)_2_ diffusive surface layer showed a high level of resistance to *E. coli* colonization in comparison with NiTi, which indicates the possibility of using this surface layer in the modification of NiTi implants’ properties.

## 1. Introduction

NiTi shape memory alloys are increasingly being used in medicine for the production of cardiac implants such as stents, blood clotting filters, implants for closing the ventricular septum in the heart [1,2] or as bone implants (e.g., bone clamps and orthodontic wires) [3,4]. The main problem associated with the use of NiTi shape memory alloys, especially in the case of long-term use implants, is the transition of nickel from the alloy to the tissues surrounding the implant, which is referred to as metallosis and can lead to allergenic, cytotoxic and even carcinogenic effects [5,6]. The nanometric oxide layers formed spontaneously on the surface of the NiTi alloy, the composition of which depends on the conditions of the implant manufacturing process, do not provide sufficient protection against metallosis [7,8]. Therefore, different methods of treating the surface of NiTi alloys are used, which are aimed at both limiting the transfer of nickel to the biological environment as well as shaping the properties of implants, depending on their intended use [9,10,11,12,13]. According to our earlier studies [10,12] and the literature data [6,9,14,15,16], TiO_2_ titanium oxide plays an important role in shaping these properties, including the biological properties of NiTi shape memory alloys. Oxide layers can be produced primarily by electrochemical oxidation, gas oxidation or low-temperature glow discharge plasma [6,12,16,17,18]. They are also used as interlayers for coatings (e.g., carbon layers such as C:N:H; in other words, amorphous hydrogenated nitrogen-containing carbon characterized by good hemocompatibility [19] or hydroxyapatite coatings for bone implants [16,20]. TiO_2_ titanium oxide, in particular the polymorphic variant of TiO_2_ titanium oxide (i.e., rutile with a nanocrystalline structure), increases biocompatibility when in contact with vascular endothelial cells [20,21]. TiO_2_ titanium oxide also plays a fundamental role in limiting metallosis if applied in a homogeneous, several dozen nanometer-thick surface layer, which is possible in the case of treatment in low-temperature glow discharge plasma [22]. The glow discharge-assisted oxidation method allows for production of titanium oxide surface layers on NiTi and titanium alloys, which has been proven to be biocompatible and can be used to enhance the properties of bone and cardiac implants. Throughout modification of technological process parameters like the temperature, time, composition of the gaseous mixture and pressure in the reaction chamber, it is possible to produce diffusive oxide surface layers of controlled phases and chemical compositions, microstructures and surface topographies [12,23,24]. Hence, this article focuses on the influence of the cathode sputtering phenomenon on the microstructure and properties of a titanium dioxide surface layer produced on NiTi shape memory alloy using the low-temperature plasma oxidation process.

## 2. Materials and Methods

### 2.1. Specimen Preparation

NiTi shape memory alloy (50.8% at. Ni-Ti balance) was used in this study. Samples of ϕ14 mm in diameter and 1 mm thick were mechanically ground using sandpapers graded up to 1200 and cleansed in acetone in ultrasonic washer. Low-temperature plasma oxidation processes were carried out in two variants: (1) oxidation using Ar + O_2_ (80% vol.) and (2) Ar + air (80% vol.) with a preliminary cathode sputtering process in an Ar + N_2_ (1:1) atmosphere. In both variants, a 290 °C temperature and 1.6 hPa pressure were used in a so-called dynamic vacuum (i.e., in a continuous stream of gas flowing through the working chamber). Plasma oxidation in the first variant of the process was carried out for 30 min, while in the second variant it was performed for 20 min, but before oxidation, the cathode sputtering process was applied at reduced pressure in the working chamber, specifically 0.50 hPa for 10 min in argon-nitrogen plasma. Heating of treated samples up to 290 °C was carried out in pure Ar (5N) and hydrogen (10% vol.) in 2.0 hPa pressure. The reasoning behind using a 290 °C temperature was that, as shown in our previous study [10], this is the maximum temperature where formation of an NiTi intermetallic intermediate sublayer does not occur, which could negatively influence the shape memory properties of NiTi alloy.

### 2.2. Microstructural and Chemical Composition Analysis

The investigations were carried out using a TECNAI SuperTWIN (200 kV) FEG transmission electron microscope (TEM) (FEI, Eindhoven, The Netherlands) capable of working in scanning mode (STEM). The nanoscale local chemical analysis was performed with an integrated EDAX energy dispersive microanalysis (EDS) system (EDAX, Tilburg, The Netherlands). Microstructure observations were performed in bright field (TEM/BF) and high resolution (TEM/HR) modes, as well as using a high angle annular dark field detector (STEM-HAADF) (Fischione Instruments, Inc., Hanau, Germany). The latter was used for setting the position of the EDS line profiles. The thin foils were prepared with an FEI Quanta 200 3D focused ion beam (FIB) (FEI, Eindhoven, The Netherlands) equipped with a lift-out system supplied by Omniprobe (Omniprobe Inc., Dallas, TX, USA). The coating surface was separated from the platinum masking bar (used in the FIB lamella preparation procedure) with an additional carbon layer. The mesoscale linear distribution of the elements—oxygen, nitrogen, titanium and nickel—in the surface layers was measured by secondary ion mass spectrometry (SIMS) analysis (Cameca IMS6F) (Cameca, Quai des Gresillons, France). Ionic beam etching with an 800 eV Ar+ ion beam was carried out.

### 2.3. Surface Topography

The surface topography of the samples was analyzed using a WYKO NT 9300 optical profilometer (Veeco, Plainview, NY, USA) and a Veeco AFM (Digital Instruments, Santa Barbara, CA, USA) equipped with a Multimode IIIa SPM Controller controller. An ACSTA AppNano tip in tapping mode (Applied Nanosctructures, Inc., Mountain View, CA, USA) was used in this study. The stereometric parameters were calculated from 480 × 640 µm areas in the case of the optical profilometer and 10 × 10 µm for the AFM. For each sample, three different areas were analyzed, and the mean and standard deviation were calculated.

### 2.4. Bacterial Colonization

The tested samples were placed in separate vessels containing a medium composed of NaCl (1%), bactopeptone (1%) and yeast extract (0.5%) with a pH of 7.0. The samples were completely submerged. A small number of *E. coli* bacteria (LGC Standards, Middlesex, UK) (2 × 103) was then added. Incubation was carried out at 37 °C for 24 h. Immediately after the end of incubation, the samples were rinsed with distilled water to remove nonadherent cells and then gently dried. The *E. coli* cells present on the surface of the samples were stained with bis-benzidine and propidium iodide in order to visualize them microscopically and distinguish live and dead cells. For this purpose, 10 µL of each dye (100 µg/mL stock solution) were applied to the test surfaces, and the dyes were allowed to penetrate the cells for 5 min at 28 °C in the dark. After that, the samples were watched using an Olympus GX 71 fluorescence microscope (Olympus, Tokyo, Japan), and the obtained images were recorded using a CCD camera (DC 73) (Olympus, Tokyo, Japan). Image acquisition was performed using analySIS DOCU software (analysis 1.9, Olympus, Tokyo, Japan) and Image J software (1.53c, Wayne Rasband NIH, Bethesda, Rockville, MD, USA), and a cell counter plugin (ver.2, Kurt De Vos, University of Sheffield, Sheffild, UK) was used to count the bacteria. Nine samples of each material were tested in three independent experiments. Six randomly selected areas were analyzed on each studied surface. The results are presented in the charts as the mean ± SD [25].

## 3. Results

**Figure 1:**
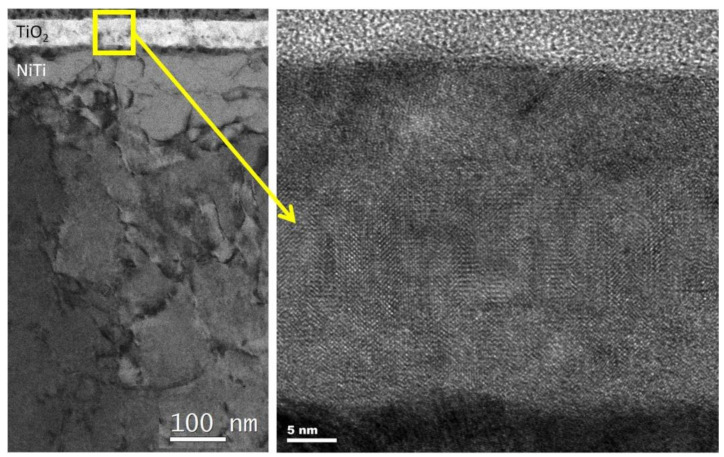
Transmission electron microscope (TEM) microstructure of a layer of nanocrystalline titanium oxide (TiO_2_) produced under glow discharge conditions in argon + oxygen plasma (Ar/O_2_ = 1/4).

**Figure 2:**
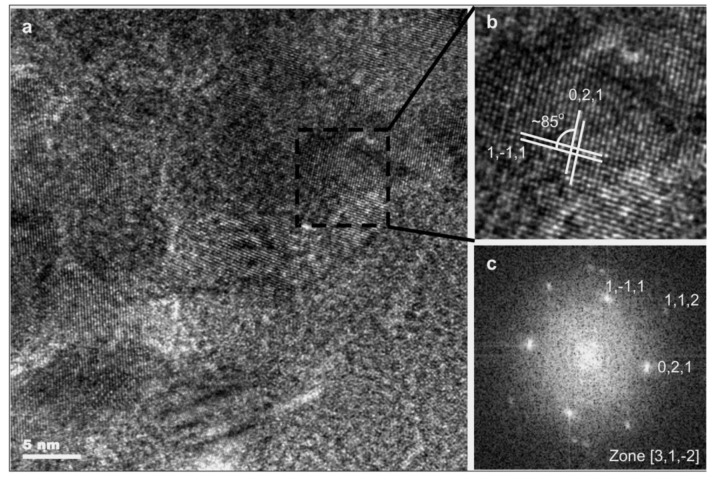
TEM microstructure (**a**,**b**) and Selected Area Electron Diffraction SAED (**c**) of a nanocrystalline titanium oxide (TiO_2_) produced under glow discharge conditions in argon + oxygen plasma (Ar/O_2_ = 1/4).

Figure 1 shows the microstructure of a titanium oxide layer produced on NiTi alloy in an atmosphere of argon + oxygen in the glow discharge. The near interface areas of the substrate are slightly darker due to the presence of dislocations caused by pretreatment polishing. The titanium oxide grown above it was dense and free from any voids or porosity, as well as relatively coarse crystalline (see the High Resolution Electron Microscopy HREM image obtained from the square marked in Figure 1). Higher up and away from the interface, the microstructure of the oxide was still compact but significantly refined (i.e., dominated with equiaxed grains from 5 nm to 10 nm (Figure 2)). The accompanying fast Fourier transform (FFT) of the HREM image of one of these crystallites confirmed that the layer was formed by a rutile phase.

**Figure 3:**
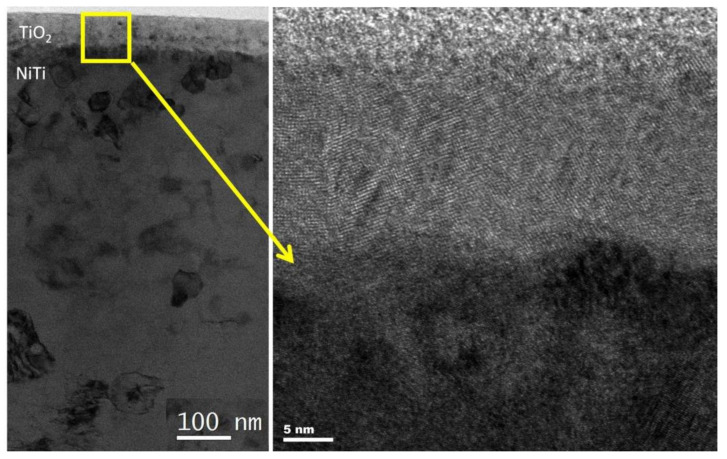
TEM microstructure of titanium oxide produced under glow discharge conditions in an argon + air reactive atmosphere (Ar/air = 1/4) with initial cathodic sputtering in argon-nitrogen plasma (Ar/N_2_ = 1/1).

Figure 3 shows a layer of titanium oxides obtained after 20 min in the same technological parameters in the Ar + air plasma, but after 10 min of cathodic sputtering. In this case, the material grown on the NiTi substrate was also fully dens but heavily defected and fine crystalline (probably due to the admixture of TiN). A coarse crystalline rutile occupied the middle part of the grown layer. The near surface area was again formed by fine crystalline material.

**Figure 4:**
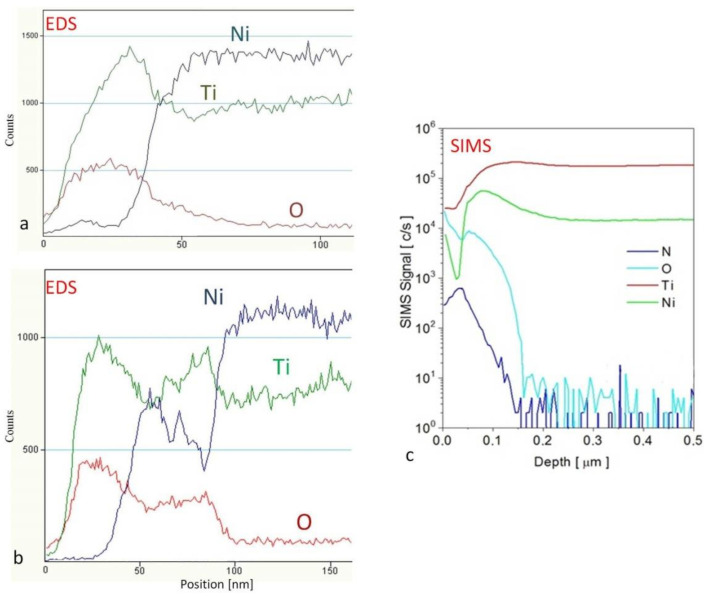
Distribution (via energy dispersive microanalysis (EDS)) of oxygen, nitrogen, nickel and titanium in the surface layers produced under glow discharge oxidation conditions without (**a**) and after initial cathodic sputtering (**b**,**c**), found using secondary ion mass spectrometry (SIMS).

The distribution of oxygen, nickel, titanium and nitrogen in the produced surface layers is shown in Figure 4.

In the case of cathodic sputtering application, a thicker layer of titanium oxide was obtained (about 80 nm), while without sputtering, the layer’s thickness was about 40 nm (Figure 4), despite the application of less chemically active air (instead of oxygen) in the reactive atmosphere during plasma oxidation. From the thermodynamic point of view, the Gibbs free energy (about 800 kJ/mol for a 300 °C temperature) favored formation of the most thermodynamically stable (in comparison to other titanium oxides) TiO_2_ oxide [26], which was confirmed in our previous studies [12,27]. It has been shown that in low-temperature plasma, TiO_2_ oxide can also be produced during the oxidation of titanium nitride during the oxynitriding process [12]. It should be noted that in plasma oxidation of NiTi alloy, high chemical affinity of the oxygen particles (i.e., atoms in statu nascendi) is not the only factor that plays a key role during formation of an oxide layer, but also prior cathodic sputtering. The sputtering phenomena has influence on the chemisorption and diffusion of active nitrogen particles (Figure 4c). Nitrogen was present throughout the thickness of the oxide layer, and due to the formation of an oxide layer and the high affinity of titanium to oxygen, nickel appeared only in the transition zone between the layer and the substrate, increasing its content with its distance from the surface. In the case of the more oxygen-rich reactive atmosphere (Ar + 20% O_2_), the highest amount of nickel was noted in the area of a lower oxygen presence. On the other hand, for prior cathodic sputtering and the nitrogen diffusion variant, this transition zone was much thicker (c.a. 30 nm (Figure 4b)). The use of cathodic sputtering before the glow oxidation process resulted in lower surface topography development of the treated NiTi alloy than in the case of only plasma oxidation (Table 1). This effect, as shown in Figure 4b,c, was related to the nitrogen and oxygen presence in the transition zone of the intermetallic Ti-Ni sublayer, which could also contribute to a reduction of residual stresses of the thicker oxide layer, thus influencing the mechanical properties and adhesion of the layer [19]. Differences in the surface roughness obtained by AFM and the optical profilometer were related to different area dimensions from which the roughness parameters were calculated. This variant of the process could also lead to the formation of a TiO_x_N_y_ surface layer. The higher thickness of titanium oxide, its topography, the nanocrystalline structure and the chemical composition all have a significant influence on the properties of treated Ni-Ti alloy and also affect other properties (e.g., wettability and free surface energy) which, on the other hand, have influence on the formation of biofilm and consequently the type of adhered cells. These relations were also discussed in our previous study on Ti6Al4V titanium alloy for blood platelet adhesion [20] and osteoblast cells in the case of nanocrystalline titanium nitride on NiTi alloy [10].

The introduction of cathodic sputtering in a nitrogen-hydrogen atmosphere before oxidation in low-temperature plasma and bombardment of the surface with nitrogen and argon ions formed in the glow discharge conditions ensured not only cleaning of the treated surface of the NiTi alloy from any adsorbed gases and oxides that had formed spontaneously during sample preparation, but due to the defects appearing in the crystalline structure, it accelerated the nitrogen and oxygen diffusion processes and contributed to a greater thickness of the Ti(O_x_N_y_)_2_ surface layer. It should be noted that during heating of the NiTi alloy in glow discharge conditions, the cathodic sputtering phenomenon was less intense due to higher pressure in the reaction chamber (2.0 hPa). The surface layer of titanium oxide-rutile, as shown in our earlier studies [12,19,21], significantly changed the properties of the NiTi, including its corrosion resistance and mechanical properties. TiO_2_ is characterized by good biological properties [19,21,28,29], including antibacterial properties [30]. That is why only Ti(O_x_N_y_)_2_ was submitted to studies on *E. coli* colonization in comparison to plain NiTi.

**Figure 5:**
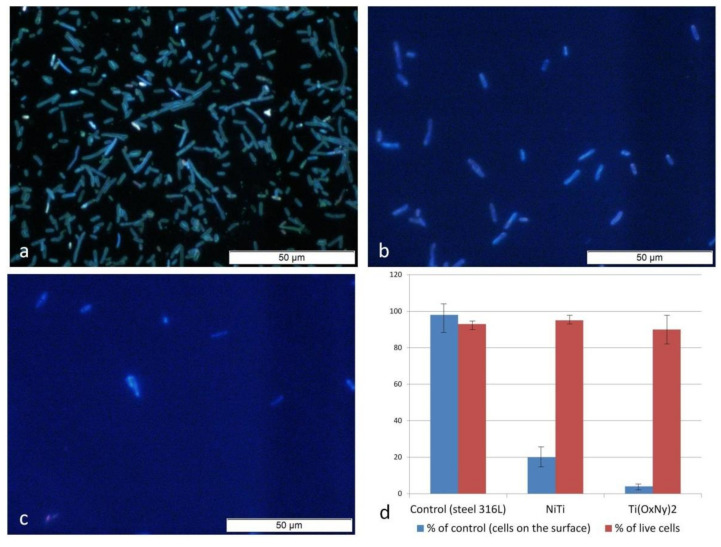
Images of *E. coli* cells cultured for 24 h on the control surface (316L steel) (**a**), the NiTi alloy surface in the initial state (**b**) and with a Ti(O_x_N_y_)_2_ surface layer (**c**), as well as the quantitative evaluation of colonization of the tested surfaces with *E. coli* bacteria in relation to the control and percentage of living cells (**d**).

All the tests for *E. coli* colonization (Figure 5) showed a high level of resistance in the polished stainless steel (AISI 316L) reference samples. For the Ti(O_x_N_y_)_2_ surface layer, the number of adhered cells was only about 5% of the reference value, while for NiTi, it was about 20% of the reference value. None of the surfaces showed any toxicity in relation to the bacteria cells. This suggests that the limited adhesion of cells to the surface was caused by a low colonization potential of the bacteria rather than the bactericidal effect of these surfaces. One study [26] showed that N–F co-doped TiO_2_ demonstrated high efficiency in its photocatalytic disinfection activity against both gram-negative and gram-positive bacteria under fluorescent light, and that metal and nonmetal co-doping techniques could also be used to improve the photocatalytic activity of TiO_2_. Therefore, it can be concluded that a Ti(O_x_N_y_)_2_ surface layer can be characterized by similar properties. Titanium oxide (TiO_2_) has been extensively explored for photocatalytic disinfection applications, which shows its advantages of high photoactivity and stability and nontoxicity [26]. It should be noted that plasma treatment processes guarantee the formation of homogeneous surface layers on titanium and its alloys on complex-shaped elements [31], which is of great significance in modifying the properties of NiTi implants, which usually exhibit complex shapes.

## 4. Conclusions

A glow discharge oxidation process carried out at 290 °C led to the formation of titanium oxide (TiO_2_) surface layers. These layers were characterized by a nanocrystalline structure. The implementation of a chemically active gas atmosphere (N_2_/Ar = 4/1) during cathodic sputtering allowed for surface activation of the treated NiTi alloy which, in the presence of active nitrogen particles (ions and in statu nascendi atoms) led to not only modification of the chemical composition produced in an argon + air atmosphere and nitrogen-doped TiO_2_ diffusive surface layer, but also the formation of almost twice as thick an oxide layer of a lower surface roughness. This is important, as glow discharge oxidation of NiTi is carried out at a process temperature below 300 °C, ensuring the preservation of the specific properties of the NiTi alloy (e.g., shape memory and superelasticity). This prevents the formation of thick intermetallic phases from the Ni-Ti system, which proceeds intensively at higher processing temperatures [27]. Therefore, by the formation of a thicker nanocrystalline Ti(O_x_N_y_)_2_ (rutile) surface layer via oxidation in low-temperature plasma, it is possible to treat complex-shaped workpieces such as implants. Investigations of NiTi alloy surface colonization before and after glow discharge oxidation processes using prior cathodic sputtering indicate that Ti(O_x_N_y_)_2_ titanium oxide does not, like TiO_2_ [30], promote bacterial adhesion on the surface, which indicates the possibility of using this surface layer in the modification of NiTi implant properties.

## Figures and Tables

**Table 1:** Surface roughness of the surface layer produced on NiTi alloy compared with material in the initial state.

Method	Material	R_a_ (nm)	R_q_ (nm)	R_z_ (nm)
Optical Profilometer	NiTi	59 ± 8	81 ± 12	1247 ± 187
	TiO_2_	173 ± 15	227 ± 27	2760 ± 331
	Ti(O_x_N_y_)_2_	144 ± 10	192 ± 12	2430 ± 315
Atomic Force Microscope	NiTi	5.6 ± 1.9	7.5 ± 2.5	88.6 ± 11.3
	TiO_2_	26.1 ± 4.8	32.9 ± 5.1	264.7 ± 31.2
	Ti(O_x_N_y_)_2_	18.8 ± 3.5	23.4 ± 4.2	184.5 ± 15.2

R_a_ is the arithmetic average of the absolute values of the profile heights over the evaluation length. R_q_ is the root mean square average of the profile heights over the evaluation length. R_z_ is the average maximum peak to valley of five consecutive sampling lengths within the measuring length.

## Data Availability

Not applicable.
